# Sparse‐Aperture Quasi‐Achromatic Spintronic Terahertz Emitter Enabled by Binary‐Phase Metasurface and Multi‐Spectral Intensity Optimization

**DOI:** 10.1002/nap2.70317

**Published:** 2026-10-08

**Authors:** Wenkai Qiu, Yongshan Liu, Yunqing Jiang, Yong Xu, Sylvain Eimer, Xiaoqiang Zhang, Weisheng Zhao

**Affiliations:** ^1^ State Key Laboratory of Spintronics Hangzhou International Innovation Institute Beihang University Hangzhou China

**Keywords:** binary phase metasurface, multi‐spectral intensity optimization, quasi‐achromatic focusing, sparse aperture, spintronic terahertz emitter

## Abstract

Terahertz (THz) technology exhibits enormous potential application in imaging and next‐generation communications. However, the lacking of compact broadband THz sources capable with focusing and wavefront manipulation capabilities remains a significant challenge for its practical application. To address this, we propose a design paradigm that combines a sparse‐aperture shared‐aperture architecture with a multispectral weighted intensity optimization method, enabling the direct construction of a Pt/W binary‐phase metasurface on a spintronic terahertz emitter. The device has an overall aperture of 2 cm and features three groups of centrosymmetric sector‐shaped sub‐apertures, each group individually optimized for specific sub‐bands. Numerical results demonstrate that the device achieves broadband quasi‐achromatic focusing over the continuous 1.0–1.5 THz band, characterized by overlapping depth‐of‐focus ranges and stabilized focal‐spot intensity at a common design plane. The spectral intensity fluctuation is suppressed to below 33%. Meanwhile, at all representative frequencies, the edge preservation index remains above 0.68, verifying the robust broadband focusing performance. This work establishes a new paradigm for active terahertz metasurface design and provides a viable pathway toward compact, efficient, and quasi‐achromatic integrated photonic systems for advanced imaging and communication applications.

## Graphical abstract

We propose a sparse‐aperture shared‐aperture design with multispectral intensity optimization, enabling a Pt/W binary‐phase metasurface directly integrated with a spintronic emitter. The device achieves quasi‐achromatic focusing across 1.0–1.5 THz, with intensity fluctuation below 33% and edge preservation above 0.68.

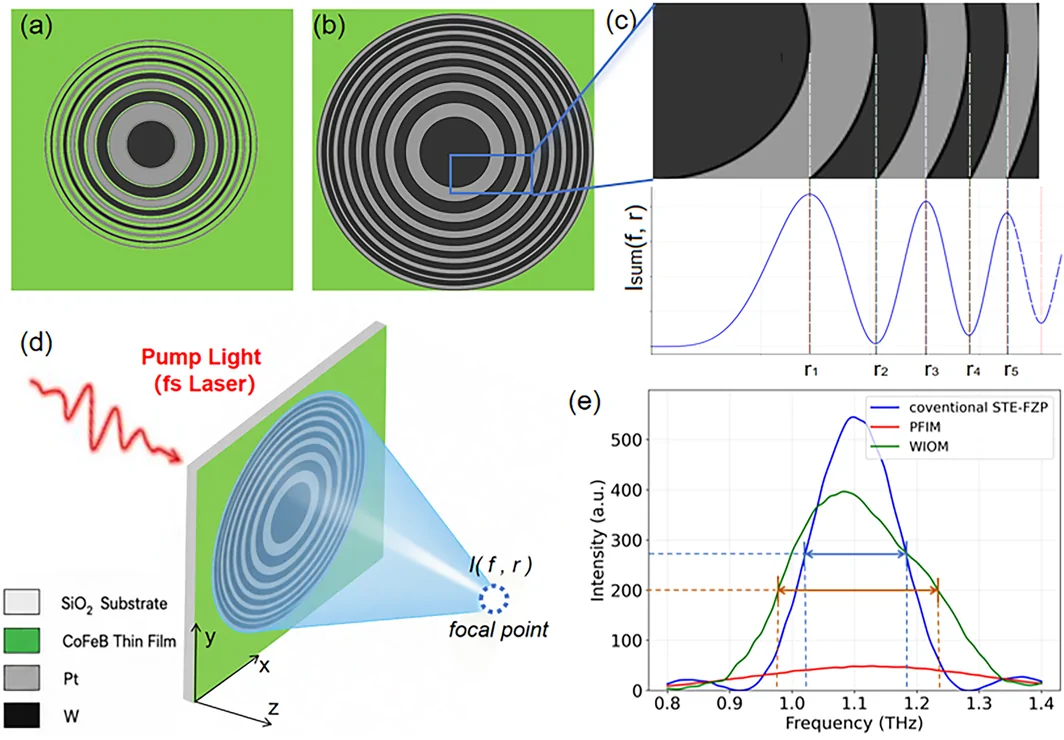

## 1 Introduction

Terahertz (THz) waves occupy the spectral range from 0.1 to 10 THz, lying between microwaves and infrared light. Their unique spectral characteristics endow them enormous application potential in nondestructive testing, biomedical imaging, high‐speed communications, and security screening [1, 2, 3]. Motivated by these prospects, extensive research efforts have been devoted to advancing THz sources and key devices of THz systems and yields notable achievements [4, 5] However, most THz sources rely on bulky external THz components for beam collimation and focusing, besides some THz elements suffer from pronounced chromatic aberration, which limits their performance in broadband and multiband applications. These drawbacks severely hindering the development of miniaturization and integration of THz systems.

In recent years, the rise of metasurface technology has provided an entirely new solution to multifunctional and integrated THz wavefront manipulation [6]. By designing artificial meta‐atoms at subwavelength scales, metasurfaces enable flexible control over the amplitude, phase, polarization, and orbital angular momentum of electromagnetic waves within an ultrathin planar form, giving rise to revolutionary THz metasurface devices [7, 8], including beam steering [9], holography [10], special beam generation. [11]. In the THz regime, both passive and active metasurfaces have flourished, with functionalities ranging from polarization conversion to multifunctional wavefront manipulation [12]. Despite these impressive advances, an inherent limitation remains, metasurfaces fundamentally require an external THz source for illumination, which introduces additional system complexity, alignment challenges, and insertion loss. This separation of source and device restricts their application in compact integrated optoelectronic systems.

Designing metasurfaces in THz sources is an effective solution to address this challenge. STEs, featuring high performance [13], low cost, and easily designed complex patterns, have become a promising approach to achieving this goal [14, 15]. Under an external magnetic field, when a femtosecond laser pulse impose on a STE, composed of a ferromagnetic/nonmagnetic (FM/NM) heterostructure, an ultrafast demagnetization effect drives a spin‐polarized current from the FM layer into the adjacent NM layer [16]. Then, an ultrafast charge current with picosecond scale is generated from the ultrafast spin current, which is due to the inverse spin Hall effect of the interface of the FM and NM layer. Then, the STE radiates a THz pulse [17]. By reversing the NM and FM layers' order or sputtering NM films with opposite spin Hall angle (e.g., Pt and W) on a FM layer an intrinsic binary phase modulation (0/π) can be directly encoded into the emitted THz wavefront. This unique property have demonstrated that STE can be combined with Fresnel zone plates (FZP) to generate focused THz beams and even focused vortex beams with different topological charges [18, 19].

Although metasurfaces have achieved remarkable progress in focusing efficiency and operating bandwidth, chromatic aberration remains a bottleneck that restricts their practical application. THz waves at different frequencies are focused at different positions, resulting in blurred imaging and signal distortion. This dispersive effect is particularly severe in multiband or broadband applications [20]. Therefore, developing achromatic THz metasurfaces that enable stable focusing at multiple wavelengths or across a broad bandwidth has become a key scientific issue urgently awaiting resolution in the field of THz photonics. In the visible and infrared bands, achromatic metalenses have achieved remarkable progress through strategies such as dispersion engineering [21], spatial multiplexing, global optimization algorithms, and utilize the natural catenary curve continuous and linear geometric phase variation [22]. In the terahertz band, several achromatic metalenses have been reported in recent years [20], including designs based on continuous dispersion phase compensation, amplitude‐modulated designs [23], a hybrid dielectric meta‐atom design [24], a photonic momentum transformation combining geometric and dynamic phases [25], and a discrete multiwavelength design combine with particle swarm optimization. The latest advances have demonstrated an ultrabroadband aberration‐free terahertz metalens via 3D printing [26]. Directly designing achromatic FZP on a STE provides a solution of source‐integrated emitter. Moreover, the aforementioned studies mostly concentrate on single‐band or limited‐band achromatic designs, involve complex parametric coupling in the structural design [27]. Faced with the increasing demand for multiband and multifunctional integration, traditional design methods based on physical intuition and empirical formulas are cumbersome and inefficient, necessitating the introduction of more efficient and intelligent design paradigms [28].

To address these challenges, we develop a multispectral incoherent intensity resonance design methodology. The first stage maximizes a multifrequency weighted total intensity using the Rayleigh–Sommerfeld diffraction integral (RSDI) at each discrete frequency and determines the Pt/W ring boundaries from the local maxima of this objective under a rising‐edge constraint. This relaxes the rigid phase‐matching conditions and achieves a 100% effective filling factor of Pt or W layer on the FM layer over the entire aperture while maintaining broadband constructive superposition at the common focus. To further widen the bandwidth, we adopt a sparse shared‐aperture architecture, the 2‐cm circular aperture is split into three centrosymmetric sector groups, and each independently optimized via the above method for a specific sub‐band. Following deep‐learning‐driven optimization of the sector‐area ratios, the focal intensity becomes relatively uniform across the entire 1.0–1.5 THz band, with the spectral intensity fluctuation suppressed to below 33% and the focusing efficiency ranging from 7.72% to 11.44%. In this work, ‘quasi‐achromatic’ is used in the practical context of broadband THz time‐domain spectroscopy, referring to the overlap of the depth‐of‐focus ranges of all frequency components at a common design plane, together with stable transverse focal profiles. This work directly embeds broadband wavefront control into an STE, offering a compact pathway toward multifunctional THz imaging and communication systems.

## 2 Design and Methods

### 2.1 Working Mechanism of the STE‐Integrated FZP

The device proposed in this study is a STE‐integrated FZP that directly radiate quasi‐achromatic focused THz waves. The core of the device is a FM/NM heterostructure, where a 4‐nm‐thick CoFeB film serves as the spin‐current generation layer and an alternating patterned structure of Pt or W, each 4 nm thick, constitutes the nonmagnetic capping layer [29]. The entire heterostructure is fabricated on a SiO_2_ substrate. Under the paraxial approximation, the outer radius *r*_*n*_ of the *n*th half‐wave zone of a conventional FZP is given by [19]:

(1) $$r_{n}=\sqrt{\frac{\lambda^{2}{\cdot n}^{2}}{4}+n\;\lambda \;F}\;\left(n=1,2,\text{…},N\right).$$

here *F* is the focal length and *λ* is the wavelength. The radii calculated from this formula precisely define the boundary positions of each annular zone composed of alternately deposited Pt or W [16]. However, this formula ensures the single wavelength of *λ* is focused at point *F*. For the goal of designing a broadband radiation source operating over a continuous frequency band *f* = [*f*_min_, *f*_max_], this single‐frequency criterion fails drastically, causing significant dispersive focal shift. The ideal zone boundaries required by different frequency components are mutually misaligned and cannot be accommodated by a single analytical expression. If a design based solely on the central wavelength is forcibly adopted, severe dispersive focal shift and efficiency degradation may occur at the band edges.

### 2.2 Phase‐Filtered Intersection and Multi‐Spectral Intensity Optimization

The core challenge in designing broadband focusing STE‐integrated FZP lies in determining the radial boundaries of the Pt/W concentric rings. We first attempted an intuitive approach—the phase‐filtered intersection method (PFIM)—to achieve multifrequency compatibility within each band [6]. For each discrete frequency *f*_*i*_ in the target band, the phase accumulated by a wave propagating from a point at radius *r* on the emission plane to the focal point *F* = 3 cm is

(2) $$\phi_{i}\left(\;r\;\right)=\frac{2\;\pi {\;f}_{i}}{c}\left(\;\sqrt{r^{2}+F^{2}}-F\;\right),$$

where *c* is the speed of light in the air [19]. The condition for the wavefront from a given point on the aperture to interfere constructively at the focal point is that its phase contribution falls within the in‐phase interval. In this work, a phase‐threshold criterion is directly applied to identify regions of constructive interference, where the phase lies within [−π/2, π/2] as the in‐phase condition. The final broadband mask is obtained by taking the Boolean intersection of the mask regions for all sampled frequencies [30]. This method suffers from a fundamental limitation: the regions satisfying the in‐phase condition for all frequencies are relatively sparse along the radial direction, leaving substantial ‘dead zones’ between the rings where neither Pt nor W is assigned to contribute, as shown in Figure 1a, resulting in a relatively low effective filling factor and a loss of overall emission efficiency.

**FIGURE 1:**
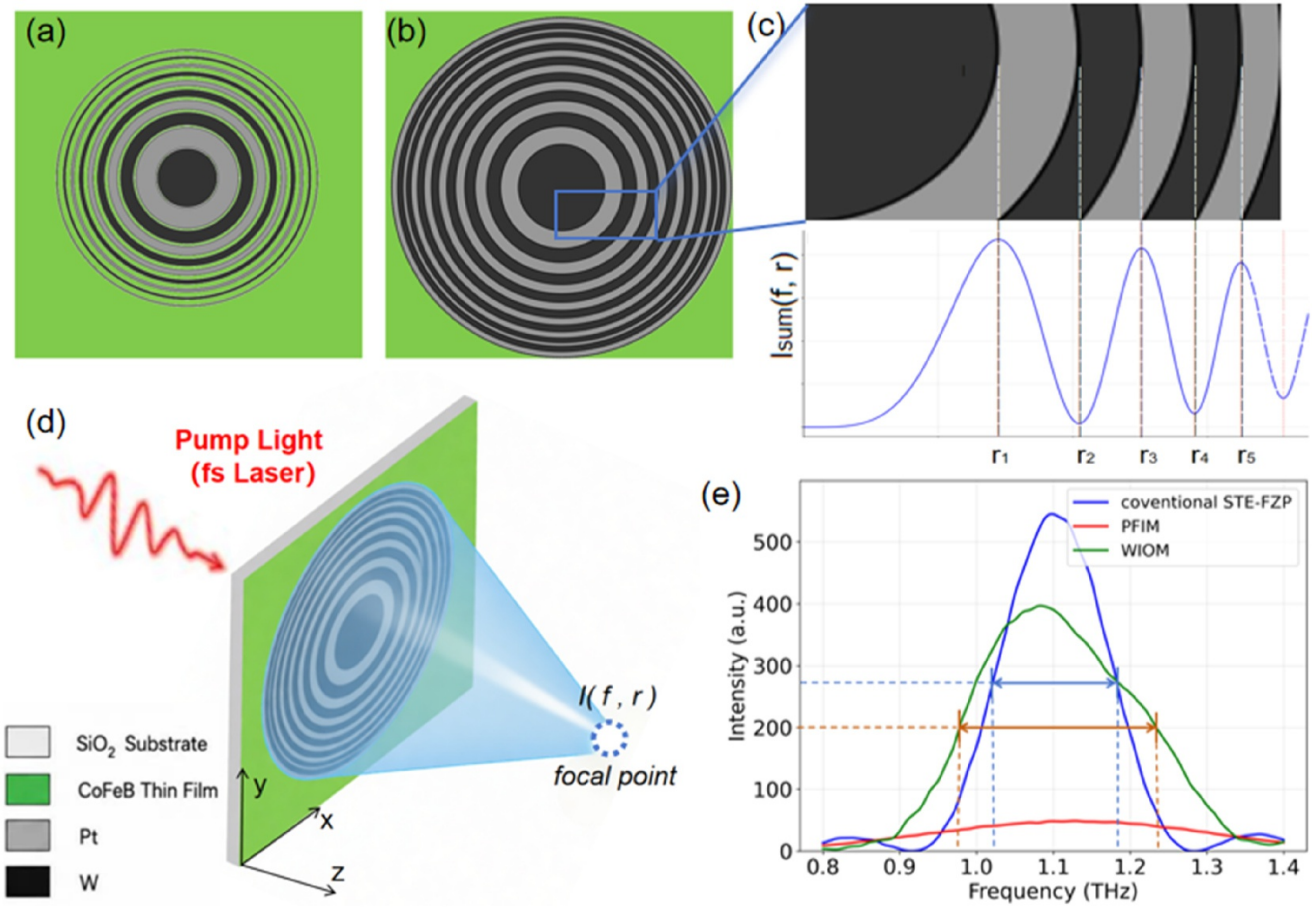
Design evolution, working principle, and spectral comparison of the Pt/W binary‐phase pattern. (a) Sparse concentric ring pattern generated by the PFIM. (b) Densely packed alternating Pt/W concentric ring array obtained by the multiband WIOM. (c) Magnified view of the central region of (b), with the corresponding radial variation of the weighted total intensity $I_{\text{sum}}\left(f,r\right)$ shown below. (d) Schematic of the device structure and working mechanism. A femtosecond laser pulse incident from the left imposes the STE‐integrated FZP designed by WIOM, generating quasi‐achromatic focused THz wave at a prescribed point. (e) Normalized emission intensity spectra (0.8–1.4 THz) at the design focal plane (*f* = 3 cm) for three configurations: a conventional STE‐integrated FZP, a STE‐integrated metasurfaces designed by the PFIM, and a STE‐integrated metasurfaces constructed by the multiband WIOM.

To overcome these inherent shortcomings, the multispectral weighted intensity optimization method (WIOM) is proposed. The design philosophy shifts from the strict in‐phase condition that enforces perfect constructive interference for all frequencies (i.e. clearly mutually exclusive across a broad bandwidth) to maximizing the weighted total intensity at the common focal point (Figure 1b). The physical basis is that the various frequency components of a STE superimpose incoherently in the far field, and the ultimate criterion for focusing performance should be the maximization of the total focal intensity rather than the individual phase matching of each frequency component. The RSDI is introduced as an accurate numerical evaluation model, and it can accurately compute the diffraction field produced by an arbitrary zone distribution at any distance. For the STE‐integrated FZP in this study, the first‐kind RSDI is adopted, whose inclination factor naturally reduces to the cos*θ* form near the normal of the emission plane. The complex amplitude at the focal point for the *i*th frequency is then given by [19].

(3) $$U_{i}\left(F\;\right)=\frac{1}{j\lambda }\cdot \iint_{\text{aperture}}U_{0}\left(\rho ,\phi \right)\;\frac{e^{jk_{i}R}}{R}\cos\;\theta \;dS,$$

where $U_{0}\left(\;\rho ,\phi \;\right)$ is the binary phase distribution on the emission plane, taking +1 for Pt regions and −1 for W regions (identical for all frequencies, as determined by the Pt/W pattern); *U*_*i*_(*F*) is the resulting complex amplitude at the design focal point *F* for the *i*th frequency sample; $R=\sqrt{r^{2}+F^{2}}$; and *k*_*i*_ is the wavenumber of frequency *f*_*i*_ and *θ* is the diffraction angle. It is worth noting that the binary‐phase approximation (+1 for Pt and −1 for W) adopted in Equation (3) is directly inherited from the established physics of Pt/W‐based spintronic emitters. As demonstrated in previous works [19, 31], Pt and W possess comparable magnitudes of spin Hall angle but opposite signs, leading to nearly identical THz emission amplitudes and a robust π phase difference. Furthermore, the inverse spin Hall effect is a nonresonant, broadband process, and the spin‐to‐charge conversion efficiency has been confirmed to be largely frequency‐independent over the 1.0–1.5 THz range. This integral model provides a high‐fidelity physical response function for the subsequent optimization. For a specific sub‐band, multifrequency weighted total intensity objective function $I_{\text{sum}}\left(\;f,r\;\right)$ at the design focal length *F* is defined as

(4) $$I_{\text{sum}}\left(\;f,r\;\right)=\sum_{i=1}^{K}w_{i}\cdot \;I_{i}\left(\;f_{i},r\;\right),$$

where *K* is the number of sampled frequencies within the band and $I_{i}\;\left(f_{i},r\right)=|\;U_{i}\;\left(\;F\;\right){\;|}^{2}$ is the focal intensity contribution of the *i*th frequency when the integration upper limit is taken up to radius *r*. The factor *w*_i_ is a spectral weight factor, determined by normalizing the measured emission spectrum of a bare STE [27], and is used for the next optimization stage to balance the focusing efficiency across frequencies, ensuring that frequency components with higher natural radiation power receive correspondingly larger weights in the optimization.

Figure 1b shows the STE‐integrated metasurfaces constructed by the multiband WIOM and Figure 1c shows the magnified view of the Pt/W ring boundary radius *r*_*m*_ relies on the local maxima and minima of $I_{\text{sum}}\left(f,r\right)$ along the radial direction. This criterion relaxes the stringent requirement of the PFIM that every single wavelength within the band must satisfy perfect constructive interference, thereby enabling all frequency components in the sub‐band to achieve constructive superposition at the focal point. After the ring boundaries are determined, adjacent rings are alternately assigned with W and Pt materials. To systematically evaluate the spectral response and focusing characteristics of the proposed design methodology, we first compared three emitter configurations at the design focal plane: the conventional STE‐integrated FZP with center frequency 1.1 THz, the STE‐integrated FZP designed by the PFIM, and the STE‐integrated FZP designed by the multiband WIOM. As shown in Figure 1e, the 3 dB spectral bandwidth broadens from 160 GHz (1.02–1.18 THz) for the conventional STE‐integrated FZP with center frequency 1.1 THz to 270 GHz (0.97–1.24 THz) for the multiband optimized metasurfaces. These results demonstrate that the multiband WIOM effectively improves chromatic focal shift of approximately 18%. Compared with the PMFI, the focus intensities are increased more than 8 times. Building on this advantage, we further examined the achromatic focusing performance of the multiband optimized metasurfaces within a band of 1.0–1.2 THz at five representative frequencies. Figure 2a,b shows the axial (*x–z* plane) and focal‐plane (*x–y* plane) normalized intensity distributions, respectively. Figure 2c,d extract the on‐axis and lateral intensity profiles. The focal spots across all sampled frequencies remains stable and symmetric.

**FIGURE 2:**
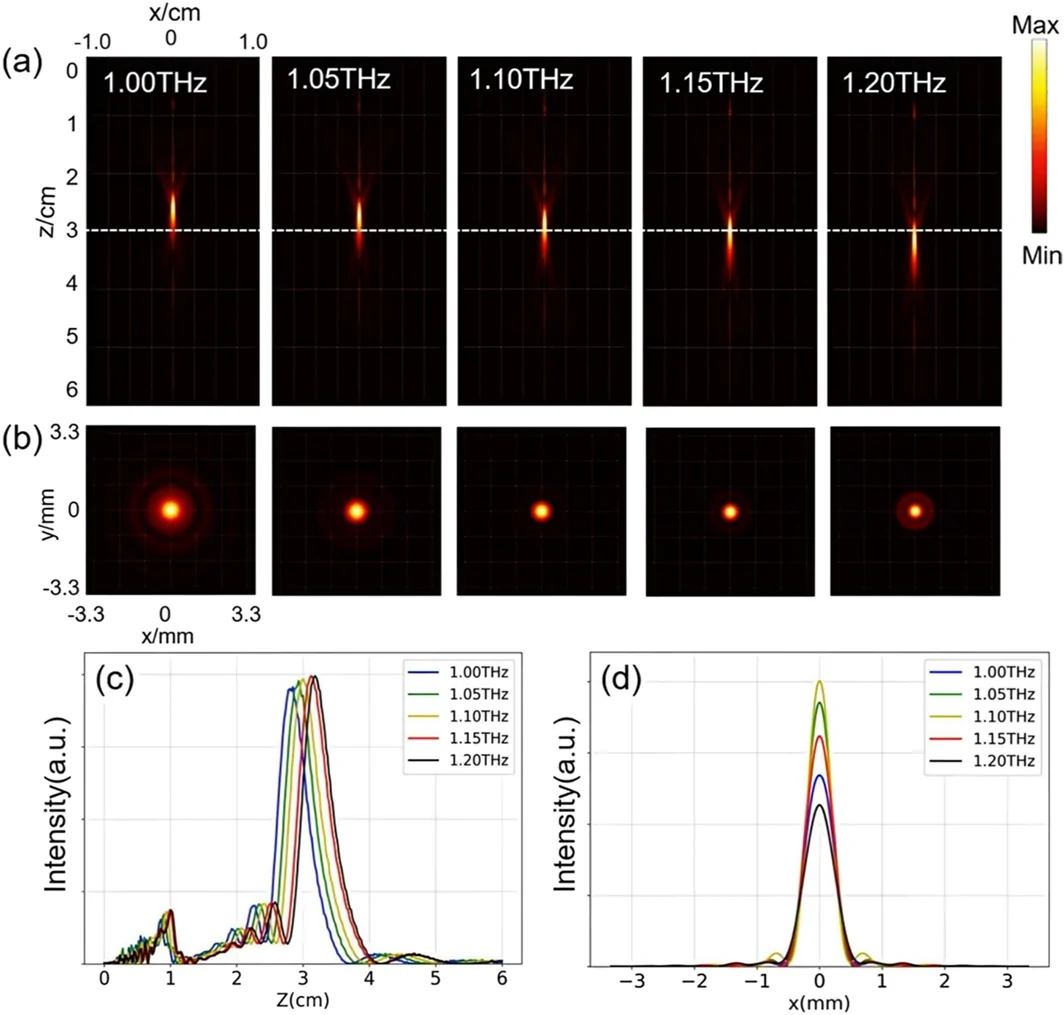
Verification of achromatic focusing within a single sub‐band (1.0–1.20 THz). Normalized intensity distributions on the *x–z* plane (a) and *x–y* plane at *z* = 3 cm (b). (c) On‐axis normalized intensity profiles extracted from panel (a). (d) Lateral normalized intensity profiles across the focal plane.

### 2.3 Sparse‐Aperture Shared‐Aperture Architecture—Tri‐Band Quasi‐Achromatic Focusing

Even with the multispectral WIOM, achieving a total bandwidth of 0.50 THz in a single full‐aperture zone plate remains challenging as shown in Figure 2b. For a relatively wide band, a zone plate designed using multispectral WIOM would cause the intensities at the band‐edge frequencies to be substantially lower than those at the center frequency. To overcome this constraint, this study further adopts a sparse‐aperture shared‐aperture architecture.

As shown in Figure 3a, the total aperture *D* = 20 mm is equally divided into nine sectors with a central angle of 40°, which are grouped into three groups arranged in a centrosymmetric manner. Each sector pair independently undertakes the focusing task for one sub‐band, dividing the wide target quasi‐achromatic band into three consecutive narrower sub‐bands, the multispectral WIOM procedure described in Section 2.2 is executed independently, yielding a complete circular ring distribution, with the sub‐band division as follows:

— Sub‐band 1: 1.00–1.15 THz
— Sub‐band 2: 1.15–1.30 THz
— Sub‐band 3: 1.30–1.50 THz

**FIGURE 3:**
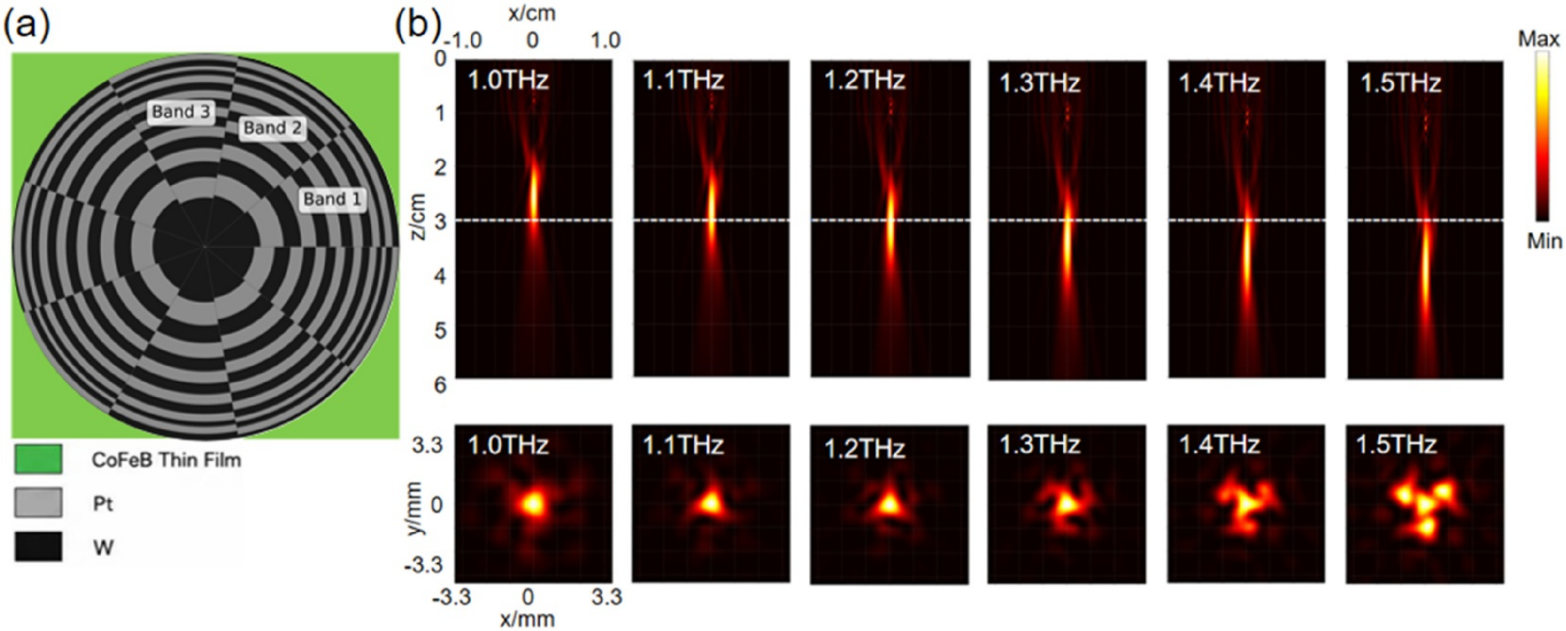
Original sparse‐aperture architecture and its achromatic focusing performance. (a) Composite metasurface pattern formed by nine 40° sectors grouped into three centrosymmetric groups. (b) Normalized intensity distributions of the final device: axial (*x*–*z* plane) and focal‐plane (*x–y* plane at *z* = 30 mm).

Considering the high‐frequency has a lower intensities, the three sub‐bands are not equally partitioned in frequency. In the low‐frequency regime, the phase accumulation is more sensitive to a given frequency deviation because the wave number is smaller, leading to a more rapid decrease of intensity at the band edges for zone boundary. For higher frequencies, the shorter wavelength reduces the diffraction‐limited spot size and provides a larger tolerance for phase mismatch at the band edges. As shown in Figure 3b, this architecture offers dual advantages. First, each sector only needs to independently correct chromatic aberration within a narrow band, not an original relative bandwidth of 40% for the entire aperture, resulting in the difficulty of achromatic correction is significantly reduced. After splicing, the total bandwidth is extended to 0.5 THz. Second, the sectors belonging to the same sub‐band are arranged in centrosymmetric groups, which can effectively suppress asymmetric aberrations, such as astigmatism, so that the focal spot remains on the optical axis of the device.

### 2.4 ResNet‐Based Optimization of Sub‐Aperture Area Ratio

The multispectral WIOM in Section 2.2 solved the radial ring design problem within each sub‐band, and the sparse‐aperture architecture in Section 2.3 provided a spatial partitioning scheme for tri‐band confocal focusing. In the initial uniform allocation scheme, all nine sectors each occupy 40°. However, due to different frequency has different proportion in the generated ultrafast spin current, a uniform angular allocation inevitably leads to significant peak‐to‐valley fluctuations in the focal spot intensity across the 1.0–1.5 THz range. To minimize this spectral nonuniformity, we further takes the total angular area proportion occupied by each of the three sub‐bands as the variable to be optimized and employs a physics‐informed data‐driven deep learning inverse design approach, constructing an optimization loop that integrates a differential evolution algorithm, a global grid search algorithm, and an embedded ResNet [32]. The structure of the ResNet is shown in the corresponding Figure 4a.

**FIGURE 4:**
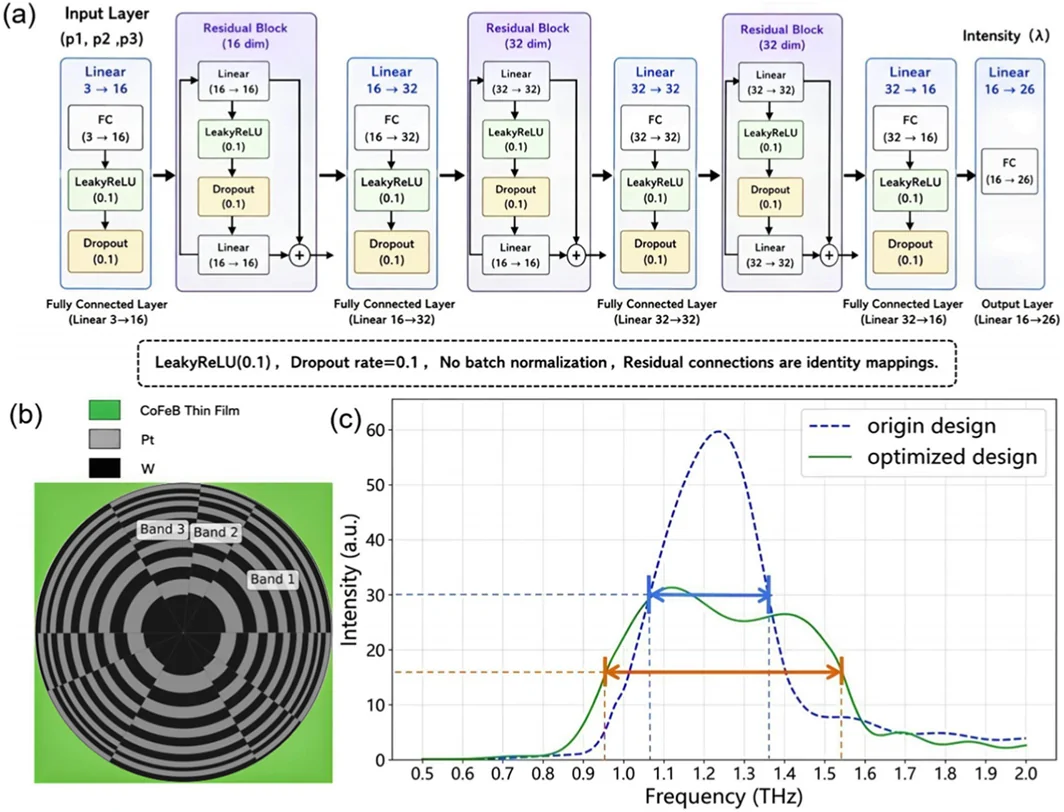
ResNet‐driven sub‐aperture area‐ratio optimization and resulting spectral uniformity. (a) Architecture of the ResNet surrogate model: the network accepts three area‐ratio parameters (*P*_1_, *P*_2_, and *P*_3_) and outputs the focal intensities at 26 uniformly sampled frequencies. (b) Optimized composite metasurface pattern after splicing the three groups of centrosymmetric sectors with the area ratios determined by the hybrid optimization. (c) Comparison of the focal‐spot peak intensity spectra (1–1.5 THz) before and after ResNet optimization.

The optimization configuration is defined as follows:

1. Input: a vector (*P*_1_, *P*_2_, *P*_3_) representing the area proportions of sub‐bands 1, 2, and 3 in the total aperture, satisfying the constraint *P*_1_ *+P*_2_ *+P*_3_ = 1.
2. For each candidate set of area proportions, the specific angles of the nine sectors are assigned. Subsequently, the ring pattern over the entire aperture is updated, and the RSDI is executed to obtain the peak focal spot intensities at *K* sampling frequencies in the 1.0–1.5 THz range.
3. The objective function is defined as the peak‐to‐valley ratio of the intensities at these *K* sampling frequencies, and the optimization goal is to minimize this function.

(5) $$L=\left(\;I_{\max}-{\;I}_{\min}\right)\;/{\;I}_{\text{mean}},$$

where *I*_max_, *I*_min_ are the maximum and minimum intensities among the *K* samples frequencies and *I*_mean_ is their average. Given the same radius for each sector, the area of the sub‐aperture is positively correlated with the sector angle. The input consists of the three area‐ratio parameters, and the output is the predicted loss value along with its gradients with respect to the parameters. The introduction of ResNet serves to verify the scalability of this method and provide a concrete implementation example of a design paradigm that fuses data‐driven and physics‐based approaches.

In this process, the ResNet serves as an efficient surrogate model of the electromagnetic response. The network is trained on 10,000 samples generated by the RSDI model (with a 70%/20%/10% training/validation/test split). The training converges after 1200 epochs, achieving a coefficient of determination (*R* ^2^) of 0.979 and a mean relative error of 5.39% on the test set (see Supporting Information S1: Figure S2 for training loss curves and error distributions). This surrogate model accelerates the forward prediction by a factor of approximately 39,800× (from 22.15 s for the full RSDI calculation to 0.556 ms for ResNet inference). This acceleration reduces the differential evolution optimization runtime from approximately 62 h (using RSDI directly) to less than 6 s using the ResNet surrogate, significantly improving the efficiency of the inverse design workflow. Under the preconditions of this work, the optimized angular width of each sector for the three sub‐bands is [60.2°, 22.9°, 36.9°].

## 3 Results

### 3.1 Focal‐Plane and Axial Intensity Distributions at Multiple Frequencies

To verify the quasi‐achromatic focusing quality, the electric field intensity distributions on the focal plane (*x*–*y* plane) and the propagation plane (*x–z* plane) are calculated at six representative frequencies uniformly covering the operating band. The results are shown in Figure 5. Figure 5a displays the corresponding axial (*x–z* plane) intensity distributions. A certain deviation of each focal position from the design focal length is observed, such a focal shift after applying the optimization algorithm for area optimization to the original structure is the purpose of the optimization is to bring the intensities of all frequency components to a similar level at the design focal length. This outcome arises from the synergy between multispectral WIOM and the sparse‐aperture sub‐band partitioning, which together ensure that the intensities of all frequency components at the common focal point are maintained within a prescribed range. Figure 5b presents the focal‐plane intensity distributions. At all frequencies, a well‐defined symmetric central focal spot is clearly observed, accompanied by faint concentric side lobes arising from the periodic ring structure and the sparse‐aperture architecture. The focal spot shape and symmetry remain nearly identical across the entire band, confirming that, despite the pattern discontinuity at the sector boundaries, the centrosymmetric sector arrangement effectively suppresses astigmatic aberrations.

**FIGURE 5:**
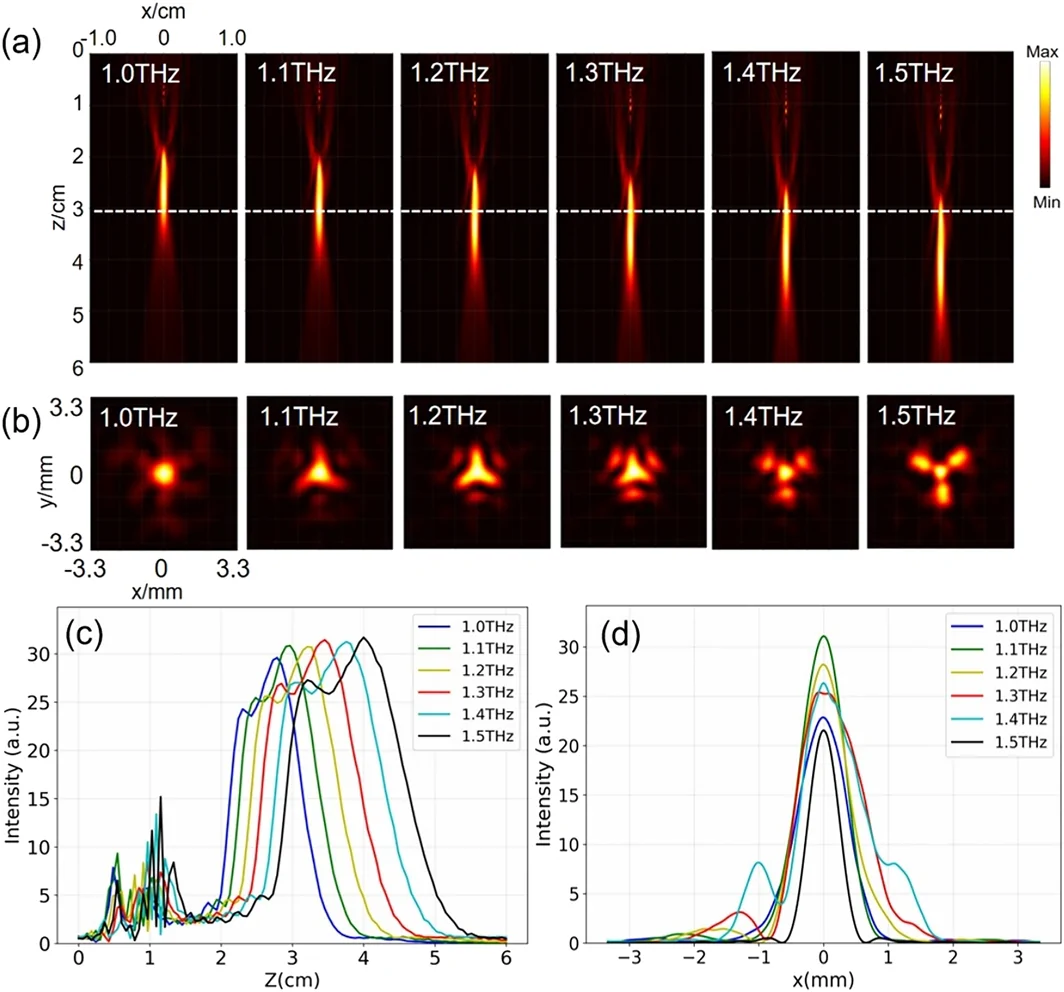
Broadband quasi‐achromatic focusing performance of the final optimized device across the 1.0–1.5 THz band. (a) Axial (*x*–*z* plane) intensity distributions at six representative frequencies. (b) Focal‐plane (*x*–*y* plane) intensity distributions at the same frequencies. (c) On‐axis normalized intensity profiles. (d) Lateral normalized intensity profiles across the focal plane.

### 3.2 Broadband Spectral Uniformity and Focusing Performance

The focal‐spot intensity at the design plane ranges reflects the broadband spectral equalization achieved through the ResNet‐driven area‐ratio optimization. Figure 4c shows the peak focal‐spot intensity spectra before and after ResNet optimization, covering an extended frequency range of 1–1.5 THz. Before optimization, due to the uniform angular allocation of the sectors for the three sub‐bands, the normalized peak and valley intensity are 60 (at 1.23 THz) and 8 (at 1.5 THz), respectively. The peak‐to‐valley intensity difference almost 87%. After ResNet optimization, the sub‐aperture area ratios were adjusted according to the optimization results, and the intensity fluctuation was reduced to below 33% (Table 1). The focal‐spot intensity uniformity is significantly enhanced.

**TABLE 1:** Simulated performance metrics of the sparse‐aperture quasi‐achromatic STE optimized by ResNet at six representative frequencies across the 1.0–1.5 THz band.

Frequency (THz)	Intensity at *z* = 3 cm plane (a.u.)	Peak intensity position (mm)	FWHM (mm)	DOF (mm)	DOF range (mm)	Focusing efficiency (%)
1.0	22.85	26.9	0.684	8.43	22.07–30.50	8.81
1.1	31.08	29.8	0.928	9.49	23.32–32.81	10.83
1.2	28.21	31.9	0.842	10.38	24.95–35.33	11.44
1.3	25.26	34.4	0.732	11.93	26.39–38.32	10.13
1.4	26.33	37.3	0.885	13.45	28.31–41.76	8.75
1.5	21.54	39.8	1.367	15.05	29.73–44.78	7.72

To quantitatively evaluate the focusing resolution, the full width at half maximum (FWHM) values and the peak position at each frequency are listed in Table 1. The measured actual peak position increases monotonically from 26.9 to 39.8 mm. The measured FWHM values range from 1.367 mm at 1.5 THz to 0.684 mm at 1.0 THz, and the focusing efficiency exhibits a narrow fluctuation of less than 4%, further confirming the favorable broadband uniformity of the quasi‐achromatic design.

We also define the DOF range for each frequency component as the axial range over which the on‐axis intensity remains above 70% of its peak value. This 70% criterion corresponds to an approximately 1.5 dB drop in signal power, which is well within the dynamic range of typical terahertz time‐domain spectroscopy and pyroelectric detection systems, and represents a practically tolerable defocus range in broadband THz imaging [33]. This gradual focal shift is an expected consequence of the design strategy adopted in this work: the optimization prioritizes intensity uniformity at the common design plane (*z* = 30 mm) and the overlap of depth‐of‐focus (DOF) ranges. As shown in Table 1, the DOF ranges of all frequency components overlap from approximately *z* = 29.73 mm to *z* = 30.50 mm, providing a common focal volume of 0.77 mm. This is a distinct advantage over a conventional STE‐FZP, which exhibits no overlapping DOF across the same band (see Supporting Information S1: Figure S1). The focusing efficiency is calculated as the power ratio within the Airy disk to the total power emitted from the metasurface. As summarized in Table 1, the efficiencies across the six frequencies remain stable between 7.72% and 11.44% with a narrow fluctuation. This spread validates that our broadband quasi‐achromatic strategy maintains highly uniform energy concentration despite the inherent efficiency trade‐off of binary‐phase and wideband designs.

### 3.3 Visual Verification and Quantitative Evaluation of Broadband Focusing Performance

A THz focused lens is widely used in the field of THz imaging. To qualitatively evaluate the imaging capability of our optimized device, we performed a numerical imaging simulation based on the point spread function (PSF). Since the spintronic THz emitter generates phase‐stable pulses across its aperture upon femtosecond laser excitation, the emitted fields from different spatial positions maintain a fixed phase relationship determined by propagation path differences. Consequently, the imaging process is modeled as a coherent convolution of the object's complex amplitude with the complex‐amplitude PSF of the device, followed by taking the squared modulus to obtain the intensity distribution. The complex‐amplitude PSF at each frequency is computed directly from the Rayleigh–Sommerfeld diffraction integral (Equation (3)), which inherently preserves the phase information of the focused field [34]. The imaging process is thus expressed as follows:

(6) $$I_{\text{image}}\left(x,y;f_{i}\right)={\left|U_{\text{object}}\left(x,y\right)⊗\text{PSF}\left(x,y;f_{i}\right)\right|}^{2},$$

where $I_{\text{image}}$ is the intensity distribution at the image plane, $U_{\text{object}}$ is the complex amplitude transmission of the object (here a binary amplitude pattern of the letters ‘THz’), and PSF is the complex‐amplitude at frequency $f_{i}$. The object is assumed to be placed immediately after the emission surface, and the image is evaluated at the common design focal plane *z* = 30 mm. This convolution model assumes fully spatially coherent illumination, which is a reasonable first‐order approximation for a single‐pulse spintronic emission process. The detailed methodology and its limitations are further discussed in the Supporting Information.

Figure 6a shows the corresponding convolution images at the six representative frequency points. As can be seen from the results, the “THz” letters are resolvable at all frequencies. For pixels in the edge region of the object plane, part of their PSF neighborhood extends beyond the object boundary, causing these pixels to appear darker in the convolution result. The object size selected in this work is 10 mm; therefore, edge vignetting will naturally appear in the simulation results. The relatively uniform and clear imaging quality across the 1.0–1.5 THz range verifies the quasi‐achromatic capability of the device. In practice, a spintronic terahertz emitter generates broadband pulses with a finite spatial coherence area; the discernibility of the “THz” letters across the entire band indicates that the structure can provide robust focusing quality over its entire operating bandwidth.

**FIGURE 6:**
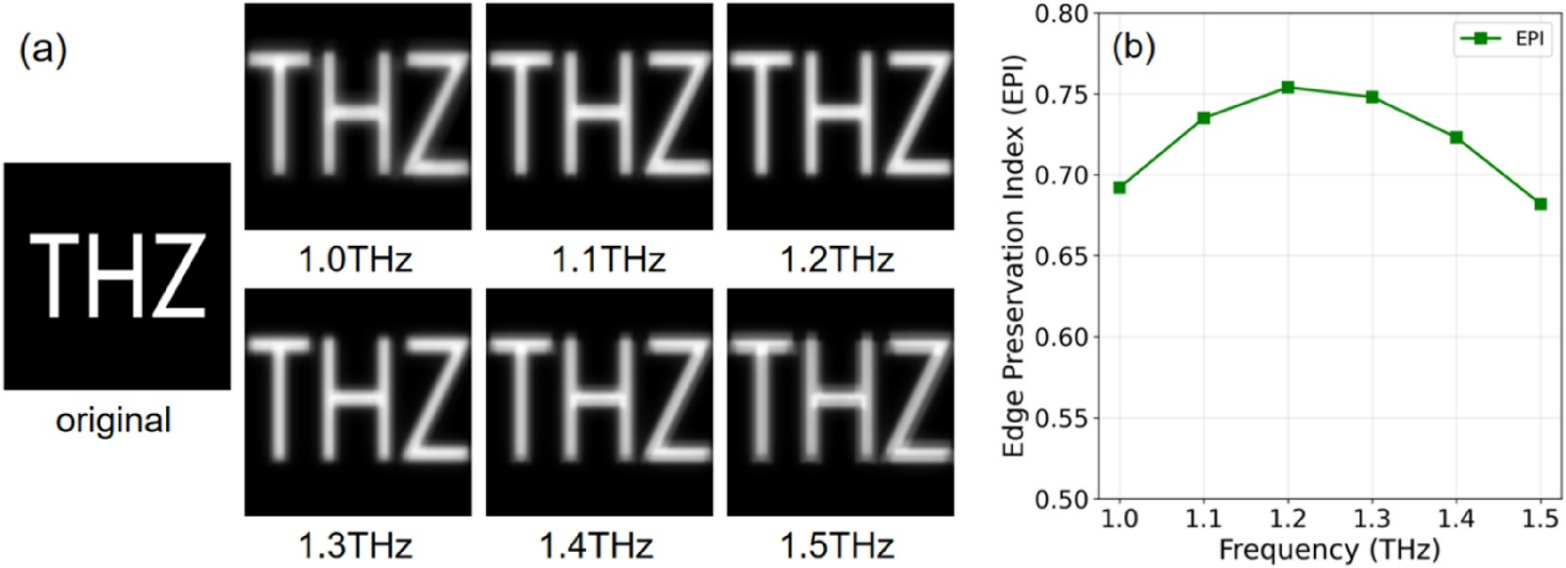
Visual verification and edge‐preservation evaluation of broadband focusing performance. (a) Simulated convolution images of a binary ‘THz’ amplitude object using the PSF at the six representative frequencies. (b) EPI as function of frequency computed from the ratio of Sobel gradient magnitudes between the convolved and original object images.

We adopt the edge preservation index (EPI) to numerically compare the degree of edge preservation between the original object pattern and the convolved image [35]. The EPI typically takes values in the range [0, 1], with values closer to 1 indicating better preservation of edge structures. The EPI results are shown in Figure 6b. The EPI values at all representative frequency points remain stable at 0.68 and above. The smoothing effect of the convolution kernel on high‐frequency details is thereby enhanced, resulting in a slight decrease of EPI without precipitous degradation. Two quantitative metrics of FWHM and EPI demonstrate that the device is capable of delivering recognizable quality imaging results over the entire 1.0–1.5 THz band.

### 3.4 Discussion

Several observations merit further discussion. First, the elongation of the focal spot observed at the highest frequency (1.5 THz) in Figure 5a is consistent with the reduced effective numerical aperture of the corresponding sector on the metasurface and the periodic sector geometry of the device surface, falling within the expected range of diffraction‐dominated behavior. Second, relatively noticeable concentric side lobes can be observed in the focal‐plane maps. This phenomenon originates from the periodic ring structure itself and the discontinuity of the sparse aperture. Through further simulation experiments, dividing each sub‐aperture corresponding to a sub‐band into more sectors arranged center symmetrically can effectively alleviate such phenomena without significantly affecting the on‐axis field intensity.

For any frequency component within the band in Figure 4c, the axial field distribution of the device evolves from a single‐peak to a double‐peak structure, with a relatively gentle variation of the terahertz intensity in the transition region between the two peaks. Analysis indicates that this evolution is a strategy adopted by the optimization algorithm which forcibly reorganizes the originally diffuse stray energy into adjacent secondary foci, signifying a pronounced difference in design paradigm between data‐driven methods and physics‐rule‐based methods.

Finally, we discuss the experimental feasibility of the proposed design. The 2‐cm‐scale device is well within current fabrication capabilities. For instance, Chen et al. [36] have experimentally demonstrated a 20 mm × 20 mm spintronic coding‐surface focusing lens using the same Pt/W binary‐phase scheme. The required nanometer‐scale FM/NM multilayers can be routinely deposited on 4‐inch wafers by magnetron sputtering, and the 2 cm aperture can be uniformly pumped by expanding the femtosecond laser beam to a 30 mm diameter as demonstrated in recent experiments [37]. The external magnetic field can be supplied by a compact Halbach array. Although our current work is a numerical design study, these references confirm that the proposed device architecture is experimentally realizable with existing technologies.

## 4 Conclusion

In summary, we have proposed and numerically verified a sparse‐aperture quasi‐achromatic spintronic terahertz emitter that directly integrates broadband focusing functionality into the radiation source plane, achieving a more compact and efficient dispersion control mechanism (broadband quasi‐achromatic focusing). By exploiting the comparable magnitude but opposite sign of the spin Hall angles of Pt and W, the device encodes an intrinsic binary phase modulation onto the emitted terahertz wavefront, thereby eliminating the need for external illumination or the fabrication of complex dielectric meta‐atom structures. To achieve broadband quasi‐achromatic focusing, multispectral WIOM replaces the stringent single‐frequency phase‐matching condition of conventional FZP with the criterion of maximizing the weighted total intensity, achieving a higher effective filling factor across the full 2 cm diameter aperture; in the angular dimension, the sparse‐aperture shared‐aperture architecture partitions the circular area into three groups of centrosymmetric sectors, each independently optimized for a specific sub‐band, and a ResNet‐driven optimization loop further adjusts the angular proportion of each sub‐band to equalize the focal spot intensities over the entire operating band.

Numerical results demonstrate that the optimized terahertz source achieves quasi‐achromatic focusing over the continuous frequency band of 1.0–1.5 THz, corresponding to a relative bandwidth of 40%. After optimization, the peak‐to‐valley spectral intensity fluctuation is reduced to below 33%, verifying the effectiveness of the data‐driven sector allocation strategy. The imaging demonstration based on the convolution of simulated point spread functions confirms that the design can theoretically resolve objects across the entire operating bandwidth. The proposed design paradigm combining physics‐based optimization at the zone level with deep‐learning‐driven allocation at the aperture level, extends to the terahertz regime or the specific Pt/CoFeB/W spintronic terahertz emitter structure, and also can be extended to other spectral regions with spintronic or source‐integrated emitters or to spintronic terahertz emitters fabricated with other materials possessing higher emission efficiency, providing a valuable concept for the development of compact terahertz sources. We envision that this work will contribute to advancing compact multifunctional terahertz imaging probes, on‐chip spectroscopy systems, and integrated front‐ends for next‐generation wireless communications.

## Author Contributions

**Wenkai Qiu:** conceptualization, methodology, investigation, software, formal analysis, data curation, visualization, writing – review and editing, writing – original draft. **Yongshan Liu:** conceptualization, methodology, data curation, investigation. **Yunqing Jiang:** conceptualization, methodology, investigation. **Yong Xu:** conceptualization, methodology, investigation. **Sylvain Eimer:** supervision, resources, conceptualization, investigation, funding acquisition. **Xiaoqiang Zhang:** conceptualization, investigation, funding acquisition, writing – original draft, writing – review and editing, visualization, validation, methodology, software, formal analysis, project administration, resources, supervision, data curation. **Weisheng Zhao:** conceptualization, investigation, methodology, funding acquisition.

## Supporting information


Supporting Information S1

## Data Availability

The data that support the findings of this study are available from the corresponding author upon reasonable request.

## References

[1] M. Tonouchi , “Cutting‐Edge Terahertz Technology,” Nature Photonics 1, no. 2 (2007): 97–105, 10.1038/nphoton.2007.3.

[2] X. R. Li , J. X. Li , Y. H. Li , A. Ozcan , and M. Jarrahi , “High‐Throughput Terahertz Imaging: Progress and Challenges,” Light: Science & Applications 12, no. 1 (2023): 233, 10.1038/s41377-023-01278-0. PMC10504281 37714865

[3] T. Nagatsuma , G. Ducournau , and C. C. Renaud , “Advances in Terahertz Communications Accelerated by Photonics,” Nature Photonics 10, no. 6 (2016): 371–379, 10.1038/nphoton.2016.65.

[4] X. Zhang , Y. Jiang , Y. Xu , et al., “Unidirectional Spintronic Terahertz Emitters With High Efficiency,” Optics Letters 47, no. 24 (2022): 6381–6384, 10.1364/ol.476809. 36538443

[5] X. J. Wu , D. Y. Kong , S. B. Hao , et al., “Generation of 13.9‐mJ Terahertz Radiation From Lithium Niobate Materials,” Advanced Materials 35, no. 23 (2023): 2208947, 10.1002/adma.202208947. 36932897

[6] W. T. Chen , A. Y. Zhu , V. Sanjeev , et al., “A Broadband Achromatic Metalens for Focusing and Imaging in the Visible,” Nature Nanotechnology 13, no. 3 (2018): 220–226, 10.1038/s41565-017-0034-6. 29292382

[7] N. F. Yu , P. Genevet , M. A. Kats , et al., “Light Propagation With Phase Discontinuities: Generalized Laws of Reflection and Refraction,” Science 334, no. 6054 (2011): 333–337, 10.1126/science.1210713. 21885733

[8] J. W. He , T. Dong , B. H. Chi , and Y. Zhang , “Metasurfaces for Terahertz Wavefront Modulation: A Review,” Journal of Infrared, Millimeter and Terahertz Waves 41, no. 6 (2020): 607–631, 10.1007/s10762-020-00677-3.

[9] S. Peng , S. R. Li , J. Ma , et al., “All‐Dielectric Asymmetric Metagrating for Broadband and Wide‐Angle Terahertz Beam Steering With High Efficiency,” Advanced Functional Materials 36, no. 3 (2026): e12746, 10.1002/adfm.202512746.

[10] T. Liu , C. H. Dai , D. Y. Wang , C. T. Chan , and L. Zhou , “High‐Efficiency Vectorial Holography Based on Ultra‐Thin Metasurfaces,” Advanced Photonics 7, no. 5 (2025): 1–11, 10.1117/1.ap.7.5.056004.

[11] X. J. Liu , M. N. Gu , Y. Tian , et al., “Research Progress on Generating Perfect Vortex Beams Based on Metasurfaces,” Opto‐Electronic Science 4, no. 11 (2025): 250007, 10.29026/oes.2025.250007.

[12] J. Li , X. G. Lu , H. Li , et al., “Racemic Dielectric Metasurfaces for Arbitrary Terahertz Polarization Rotation and Wavefront Manipulation,” Opto‐Electronic Advances 7, no. 10 (2024): 240075, 10.29026/oea.2024.240075.

[13] Y. Q. Jiang , H. Q. Li , X. Q. Zhang , et al., “Promoting Spintronic Terahertz Radiation via Tamm Plasmon Coupling,” Photonics Research 11, no. 6 (2023): 1057–1066, 10.1364/prj.487711.

[14] T. Seifert , S. Jaiswal , U. Martens , et al., “Efficient Metallic Spintronic Emitters of Ultrabroadband Terahertz Radiation,” Nature Photonics 10, no. 7 (2016): 483–488, 10.1038/nphoton.2016.91.

[15] Y. Q. Jiang , X. Q. Zhang , Y. S. Liu , et al., “Spintronic Terahertz Emitter With Integrated Electromagnetic Control,” Chinese Optics Letters 20, no. 4 (2022): 043201, 10.3788/col202220.043201.

[16] T. Kampfrath , M. Battiato , P. Maldonado , et al., “Terahertz Spin Current Pulses Controlled by Magnetic Heterostructures,” Nature Nanotechnology 8, no. 4 (2013): 256–260, 10.1038/nnano.2013.43. 23542903

[17] T. S. Seifert , L. Cheng , Z. X. Wei , T. Kampfrath , and J. B. Qi , “Spintronic Sources of Ultrashort Terahertz Electromagnetic Pulses,” Applied Physics Letters 120, no. 18 (2022): 180401, 10.1063/5.0080357.

[18] X. Q. Zhang , Y. Xu , B. Hong , F. Zhang , A. T. Wang , and W. S. Zhao , “Generation of a Focused THz Vortex Beam From a Spintronic THz Emitter With a Helical Fresnel Zone Plate,” Nanomaterials 13, no. 14 (2023): 2037, 10.3390/nano13142037. PMC10383332 37513048

[19] X. Zhang , Y. Jiang , F. Liu , Y. Xu , A. Wang , and W. Zhao , “Focused THz Wave From a Spintronic Terahertz Fresnel Zone Plate Emitter,” Optics and Laser Technology 171 (2024): 110418, 10.1016/j.optlastec.2023.110418.

[20] K. Huang , F. Qin , H. Liu , et al., “Planar Diffractive Lenses: Fundamentals, Functionalities, and Applications,” Advanced Materials 30, no. 26 (2018): 1704556, 10.1002/adma.201704556. 29672949

[21] Y. Q. Hu , Y. T. Jiang , Y. Zhang , et al., “Asymptotic Dispersion Engineering for Ultra‐Broadband Meta‐Optics,” Nature Communications 14, no. 1 (2023): 6649, 10.1038/s41467-023-42268-5. PMC10589226 37863896

[22] M. B. Pu , X. Li , X. L. Ma , et al., “Catenary Optics for Achromatic Generation of Perfect Optical Angular Momentum,” Science Advances 1, no. 9 (2015): e1500396, 10.1126/sciadv.1500396. PMC4646797 26601283

[23] F. Zhao , Z. P. Li , X. M. Dai , et al., “Broadband Achromatic Sub‐Diffraction Focusing by an Amplitude‐Modulated Terahertz Metalens,” Advanced Optical Materials 8, no. 21 (2020): 2000842, 10.1002/adom.202000842.

[24] X. Q. Jiang , X. Chen , Z. C. Bin , et al., “Large‐Aperture Polarization‐Independent Broadband Achromatic All‐Dielectric Metalens for Terahertz Focusing,” Advanced Science (2026). 10.1002/advs.74515 PMC13137821 41703960

[25] Y. H. Guo , S. C. Zhang , M. B. Pu , et al., “Spin‐Decoupled Metasurface for Simultaneous Detection of Spin and Orbital Angular Momenta via Momentum Transformation,” Light: Science & Applications 10, no. 1 (2021): 63, 10.1038/s41377-021-00497-7. PMC7994415 33767137

[26] J. Chen , S. X. Huang , K. F. Chan , G. B. Wu , and C. H. Chan , “3D‐printed Aberration‐Free Terahertz Metalens for Ultra‐Broadband Achromatic Super‐Resolution Wide‐Angle Imaging With High Numerical Aperture,” Nature Communications 16, no. 1 (2025): 363, 10.1038/s41467-024-55624-w. PMC11699036 39753564

[27] X. Q. Zhang , R. M. Li , Y. Q. Jiang , et al., “Dual‐Band Achromatic Wavefront Control of Spintronic Terahertz via Cascaded Metasurfaces,” Optics Letters 50, no. 7 (2025): 2175–2178, 10.1364/ol.554430. 40167674

[28] X. Q. Zhang , P. Vallobra , Y. Xu , X. Y. Lin , and W. S. Zhao , “Photoinduced Picosecond‐Scale Radial Current for Generating and Converting Spin Textures,” Nanophotonics 15, no. 7 (2026): e70060, 10.1002/nap2.70060.

[29] Y. S. Liu , Y. Xu , A. Fert , et al., “Efficient Orbitronic Terahertz Emission Based on CoPt Alloy,” Advanced Materials 36, no. 36 (2024): 2404174, 10.1002/adma.202404174. 38896111

[30] F. Presutti and F. Monticone , “Focusing on Bandwidth: Achromatic Metalens Limits,” Optica 7, no. 6 (2020): 624–631, 10.1364/optica.389404.

[31] X. Q. Zhang , W. K. Qiu , Y. Q. Jiang , et al., “Tri‐Band and One‐Foci Sparse‐Aperture Achromatic Spintronic Terahertz Fresnel‐Zone‐Plate Emitters by Deep Learning,” Optics Communications (2025): 592, 10.1016/j.optcom.2025.132239.

[32] M. Shafiq and Z. Q. Gu , “Deep Residual Learning for Image Recognition: A Survey,” Applied Sciences‐Basel 12, no. 18 (2022): 8972, 10.3390/app12188972.

[33] Y. C. Zeng , X. L. Ge , Y. Q. Zhang , et al., “Extended Depth of Focus Metalens Toward the Entire Long‐Wave Infrared Spectrum Through Inverse Design Framework,” Optics Express 33, no. 18 (2025): 39081–39091, 10.1364/oe.570816. 40984299

[34] X. L. Zhu , H. N. Yao , J. Y. Yu , et al., “Inverse Design of a Spatial Filter in Edge Enhanced Imaging,” Optics Letters 45, no. 9 (2020): 2542–2545, 10.1364/ol.391429. 32356811

[35] F. Sattar , L. Floreby , G. Salomonsson , and B. Lovstrom , “Image Enhancement Based on a Nonlinear Multiscale Method,” IEEE Transactions on Image Processing 6, no. 6 (1997): 888–895, 10.1109/83.585239. 18282982

[36] S. Chen , H. C. Wang , J. Y. Liu , et al., “Simultaneous Terahertz Pulse Generation and Manipulation With Spintronic Coding Surface,” Advanced Optical Materials 12, no. 6 (2024): 2300899, 10.1002/adom.202300899.

[37] Z. H. Yang , X. X. Chen , J. H. Li , et al., “Intense Spintronic Terahertz Emitter for Large‐Area Imaging,” ACS Photonics 13, no. 1 (2026): 101–110, 10.1021/acsphotonics.5c01837.

