# Supplementary material for: Sparse‐Aperture Quasi‐Achromatic Spintronic Terahertz Emitter Enabled by Binary‐Phase Metasurface and Multi‐Spectral Intensity Optimization
Source: Nanophotonics. 2026 Oct 8;15(19):e70317. doi: 10.1002/nap2.70317 (PMC13647489; doi:10.1002/nap2.70317)
Supplement: Supplementary file 1 [file NAP2-15-e70317-s001.docx]

**Supporting Materials:**

**Note 1. Simulated performance of the STE-FZP designed at 1.25 THz over the 1.0–1.5 THz frequency band.**

For a binary-phase Fresnel zone plate, the theoretical maximum focusing efficiency is approximately 40.5% [1], and the conventional FZP approaches this limit (29.71%) near its design frequency.

Table S1. Simulated performance of the STE-FZP designed at 1.25 THz over the 1.0–1.5 THz frequency band.

| Frequency  (THz) | Intensity at z = 3cm plane (a.u.) | Peak intensity position  (mm) | FWHM  (mm) | DOF  (mm) | DOF range  (mm) | Focusing efficiency |
| --- | --- | --- | --- | --- | --- | --- |
| 1.0 | 20.14 | 23.5 | 0.610 | 2.41 | 22.37-24.78 | 2.92% |
| 1.1 | 43.61 | 26.3 | 1.294 | 2.71 | 24.78-27.49 | 8.37% |
| 1.2 | 509.98 | 28.4 | 0.391 | 2.99 | 27.21-30.20 | 29.71% |
| 1.3 | 576.95 | 31.3 | 0.366 | 3.08 | 29.68-32.76 | 27.38% |
| 1.4 | 62.25 | 33.7 | 1.489 | 3.30 | 32.05-35.35 | 6.10% |
| 1.5 | 41.05 | 36.1 | 2.222 | 3.47 | 34.49-37.96 | 1.51% |

The 70% peak-intensity criterion used here to define the DOF range has been adopted in recent metalens design studies and corresponds to an approximately 1.5 dB signal-power drop, which is well within the dynamic range of typical THz-TDS detection systems [2]. The overlapping DOF ranges presented in Tables 1 from manuscript and Table S2 illustrate a key distinction between our WIOM design and a conventional FZP. For the conventional FZP, the DOF ranges at adjacent frequencies are disjointed (29.68–32.76 mm at 1.3 THz vs. 32.05–35.35 mm at 1.4 THz), meaning that no single detector position receives in-focus contributions from all frequency components simultaneously. In contrast, the WIOM design ensures that all DOF ranges overlap between 29.73 mm and 30.50 mm, providing a 0.77 mm common focal volume. This is particularly advantageous for THz time-domain spectroscopy systems with fixed detector positions.

Figure S1. Comparison of axial (x-z plane) normalized intensity distributions. A conventional STE-integrated FZP designed at 1.25 THz. The conventional FZP exhibits a sharp focus near its design frequency but collapses at the band edges, whereas the WIOM design maintains stable focusing performance across the entire 1.0–1.5 THz band.

**Note 2. Resnet training details and performance metrics**

1). Structure of data samples

Samples: 10,000 (RSDI-generated)

Training/Validation/Test split=70% / 20% / 10%

Training epochs = 1200 (converged)

2). Convergence behavior

The training and validation loss curves show that both decrease rapidly within the first 200 epochs and stabilize after approximately 600 epochs.

3). Prediction accuracy

On the held-out test set (1000 samples), the ResNet achieves:

R² (coefficient of determination) = 0.979

Mean absolute error (MAE) = 0.012

Root mean square error(RMSE) = 3.1183

Mean Relative Error(MRE) = 5.39%

4). Computational acceleration

Full RSDI calculation: 22.15 s (baseline for each set of data)

ResNet inference: 0.556 ms ~39,800×

Figure S2: (a) ResNet training and validation loss curves. (b) Relative prediction error distribution of ResNet on test samples. (c) R² value of the ResNet at each frequency point.

To benchmark the ResNet-assisted optimization against direct physics-based optimization, we performed a comparative study using the same differential evolution algorithm with two different forward models: (i) the full RSDI calculation (22.15 s per evaluation) and (ii) the ResNet surrogate (0.556 ms per evaluation). Both methods converged to nearly identical optimal angle parameters: the ResNet-assisted result yielded [60.2°, 22.9°, 36.9°], while the direct RSDI-based optimization yielded [60.5°, 22.6°, 36.9°]—a maximum deviation of less than 0.5°. This confirms that the ResNet surrogate introduces negligible error. However, the computational cost differed dramatically: the RSDI-based optimization required 61.8 hours for 10,000 iterations, whereas the ResNet-assisted optimization completed in 5.7 seconds—an effective speedup of approximately 39,000× when accounting for the total optimization runtime.

**Note 3. Detailed description of the imaging convolution model and its limitations.**

The imaging simulation was performed by convolving the complex-amplitude point spread function (PSF)—computed from the Rayleigh–Sommerfeld diffraction integral (Eq. (3) of the main text) with the object’s complex amplitude transmission, followed by taking the squared modulus to obtain the intensity distribution. This coherent convolution model assumes fully spatially coherent illumination, the coherent convolution model is justified by the phase-stable nature of single-pulse spintronic emission. The object was placed immediately after the emission surface, and the image was evaluated at the common design focal plane z = 30 mm. The binary amplitude pattern of the letters 'THz' was chosen with a feature size of approximately 10 mm. The coherent convolution imaging model employed in Section 3.3 is expressed as:

$\text{I}_{\text{image}}\text{(}\text{x}\text{,}\text{y}\text{;}\text{f}_{\text{i}}\text{) = }\text{I}_{\text{object}}\text{(}\text{x}\text{,}\text{y}\text{)}\text{⨂}\text{PSF}\text{(}\text{x}\text{,}\text{y}\text{;}\text{f}_{\text{i}}\text{)}$	(S1)

where $\text{I}_{\text{image}}$ is the intensity distribution at the image plane, ${\text{ }\text{U}}_{\text{object}}$ is the complex amplitude transmission of the object (here a binary amplitude pattern of the letters "THz"), and PSF is the complex-amplitude at frequency $\text{f}_{\text{i}}$.

The PSF convolution approach adopted here follows the methodology established in recent edge-enhanced imaging studies [3]. As discussed in Section 2.1 of the main text, the coherent convolution model is justified by the phase-stable nature of single-pulse spintronic emission. In the simulated images shown in Fig. 6(a), blurring and ghosting artifacts are observed at the edges of the "THz" letters. Two primary mechanisms contribute to this behavior. First, Gibbs ringing artifacts arise from the finite aperture truncation of the PSF, which introduces oscillatory sidelobes in the coherent diffraction pattern. When convolved with sharp object edges, these sidelobes produce the characteristic ringing fringes seen in the images. Second, the diffuse sidelobes and scattering background originating from the sparse-aperture architecture, the discrete sector boundaries, and the concentric ring structure of the binary-phase metasurface further contribute to the degradation of edge contrast. Since the PSF sidelobes extend beyond the immediate focal spot, they illuminate regions adjacent to object edges, causing the edge pixels to appear darker and producing visible halo or ghosting effects. These phenomena are normal and unavoidable consequences of coherent diffraction from finite, non-continuous apertures.

**Reference:**

[1] M. Born and E. Wolf: Principles of Optics, 7th (expanded) edition, Cambridge University Press, 1999.

[2] Zeng Y C, Ge X L, Zhang Y Q, Xiao S Y, Zhao F, Ran C C, Wu M J, Gan F Y, Wu J G, Yang J B. Extended depth of focus metalens toward the entire long-wave infrared spectrum through inverse design framework[J]. OPTICS EXPRESS, 2025, 33(18): 39081-39091.

[3] Zhu X L, Yao H N, Yu J Y, Gbur G, Wang F, Chen Y H, Cai Y J. Inverse design of a spatial filter in edge enhanced imaging[J]. OPTICS LETTERS, 2020, 45(9): 2542-2545.
